# Impact of *Nannochloropsis oceanica* and *Chlorococcum amblystomatis* Extracts on UVA-Irradiated on 3D Cultured Melanoma Cells: A Proteomic Insight

**DOI:** 10.3390/cells13231934

**Published:** 2024-11-21

**Authors:** Agnieszka Gęgotek, Tiago Conde, Maria Rosário Domingues, Pedro Domingues, Elżbieta Skrzydlewska

**Affiliations:** 1 Department of Analytical Chemistry, Medical University of Bialystok, Mickiewicza 2D, 15-222 Bialystok, Poland; elzbieta.skrzydlewska@umb.edu.pl; 2 Centre for Environmental and Marine Studies, Department of Chemistry, University of Aveiro, Santiago University Campus, 3810-193 Aveiro, Portugal; tiagoalexandreconde@ua.pt (T.C.); mrd@ua.pt (M.R.D.); 3 Mass Spectrometry Centre, LAQV-REQUIMTE, Department of Chemistry, University of Aveiro, Santiago University Campus, 3810-193 Aveiro, Portugal; p.domingues@ua.pt

**Keywords:** UVA radiation, melanoma, microalgae lipid extracts, proteome, 4-hydroxynonenal-protein adducts, 15-prostaglandin J2-protein adducts

## Abstract

Melanoma is one of the most malignant forms of skin cancer, characterised by the highest mortality rate among affected patients. This study aims to analyse and compare the effects of lipid extracts from the microalgae *Nannochloropsis oceanica* (*N.o.*) and *Chlorococcum amblystomatis* (*C.a.*) on the intra and extracellular proteome of UVA-irradiated melanoma cells using a three-dimensional model. Proteomic analysis revealed that UVA radiation significantly increases the levels of pro-inflammatory proteins in melanoma cells. Treatment with algae extracts reduced these protein levels in both non-irradiated and irradiated cells. Furthermore, untreated cells released proteins responsible for cell growth and proliferation into the medium, a process hindered by UVA radiation through the promotion of pro-inflammatory molecules secretion. The treatment with algae extracts effectively mitigated UVA-induced alterations. Notably, UVA radiation significantly induced the formation of 4-HNE and 15-PGJ2 protein adducts in both cells and the medium, while treatment with algae extracts stimulated the formation of 4-HNE-protein adducts and reduced the level of 15-PGJ2-protein adducts. However, both algae extracts successfully prevented these UVA-induced modifications. In conclusion, lipid extracts from *N.o.* and *C.a.* appear to be promising agents in supporting anti-melanoma therapy. However, their potent protective capacity may limit their applicability, particularly following cells exposure to UVA.

## 1. Introduction

Skin cancers are currently the most diagnosed cancers worldwide. Among them, melanoma, though less common, is the most aggressive and deadliest form. It is estimated that over 30% of patients with advanced-stage melanoma do not survive [1]. Additionally, melanoma is one of the cancers most likely to recur [2]. The aetiology of melanoma encompasses both genetic factors, such as genetic predispositions, gene mutations, phenotypic characteristics, and the presence of melanocytic or dysplastic naevi, and environmental factors, including ultraviolet (UV) radiation from solar and artificial sources such as indoor tanning [3,4]. The pathophysiology of melanoma involves the neoplastic transformation of melanocytes, epidermal cells that produce melanin in response to UV radiation. This melanin synthesis not only absorbs harmful radiation, thereby protecting cellular molecules, but also enhances the generation of reactive oxygen species (ROS), leading to oxidative stress [5]. Recent studies established a link between ROS overproduction and melanoma development [6]. These highly reactive molecules activate signal transduction pathways, such as RAS/RAF/ERK1/2, PI3K/AKT, and RAC1, which are implicated in the initiation and progression of melanoma [7]. Additionally, the oxidative stress accompanying ROS overproduction enhances lipid peroxidation, generating signalling molecules such as reactive aldehydes and prostaglandin derivatives that are crucial in pro-inflammatory signalling [8].

Emerging melanoma cells are characterised by their uncontrollably high proliferation, the ability to penetrate through skin layers, and the potential to metastasize [9,10]. These processes are further supported by the release of factors by cancer cells that stimulate proliferation, induce angiogenesis, and cause inflammation, thereby disrupting the function of healthy neighbouring cells, including skin fibroblasts and keratinocytes [11,12]. It has also been observed that during anticancer therapies, dying melanoma cells release various molecules, including signalling factors (pro-inflammatory, pro-apoptotic, or proliferation-inducing) and damaged or undigested proteins [13,14,15], which can compromise the viability of neighbouring healthy cells. Consequently, there is an ongoing need for compounds that can mitigate the toxicity of dying cancer cells to the surrounding tissue when used alongside conventional anticancer therapies.

Microalgae extracts, rich in bioactive compounds such as phospholipids, polyunsaturated fatty acids (PUFAs), polysaccharides, and vitamins, are increasingly being considered for their health-protective potential [16,17]. Their antioxidant and anti-inflammatory properties help regulate lipid metabolism and reduce the production of pro-inflammatory cytokines, including tumour necrosis factor-alpha (TNFα), interleukin-1β, and interleukin-6 (IL-1β and IL-6) [18,19,20], exhibiting anti-proliferative effects on various cancer cells, including lung, colorectal, breast, and prostate cancer, and notably, melanoma [21,22,23,24]. Furthermore, the therapeutic potential of algae biomass and extracts is being explored for various skin conditions [25,26,27]. However, the effects of these bioactive compounds on skin cancers under oxidative stress, including UV-irradiated melanoma cells, remain unclear.

Previous studies have shown that lipid extracts from microalgae such as *Nannochloropsis oceanica* and *Chlorococcum amblystomatis* possess antioxidant, anti-inflammatory, and anti-proliferative properties [28]. Therefore, this study aims to study and compare the effects of these two microalgae lipid extracts on the intra- and extracellular proteome of UVA-irradiated melanoma cells cultured in vitro in a three-dimensional (3D) model. This cell culture model was selected to mimic the multilayer development and cell–cell interactions of melanoma cells, which influence their aggressiveness in vivo [29].

## 2. Materials and Methods

### 2.1. Microalgal Material 

The microalgae *Nannochloropsis oceanica* and *Chlorococcum amblystomatis* needed for the study were grown in Guillard’s F2 medium supplemented with a mixture of magnesium salts and sodium chloride adapted to the water used [30]. The cultivation was carried out in 5-L reactors continuously exposed to light radiation (700 µmol photons·m^2^·s^−1^ for 7–15 days). The technical details of the culture performed were described earlier [20]. The obtained microalgae in an amount of approximately 50 g/L were dried (in a spray dryer) in an air stream (inlet temperature—215 ± 5 °C; outlet temperature—92 ± 3 °C). Microalgae were pulverised using a cyclone and stored in a dry and dark place and the obtained biomass was spray-dried (Allmicro-algae, Natural Products SA, Pataias, Portugal).

### 2.2. Microalgae Lipid Extracts

In order to extract lipids from the obtained microalgae, the Folch method (modified) was used [31,32]. Extraction was carried out with dichloromethane + methanol (2:1, *v*/*v*) solution, which was added to 25 mg of the obtained biomass, and the received suspension was centrifuged (670× *g*; 10 min). The supernatant was subjected to the above procedure four times. The obtained solutions were combined and dried in a stream of nitrogen, then dissolved in mixture CH_2_Cl_2_ + CH_3_OH and then centrifuged. After that Mili-Q water was added. After centrifugation (670× *g*; 10 min), phase separation occurred and the aqueous phase was extracted two more times. All the obtained organic phases constituted a lipid extract in which the lipid content was determined using the gravimetric method.

Hydrophilic interaction liquid chromatography combined with tandem mass spectrometry (MS/MS) (Q-Exactive Orbitrap mass spectrometer) (Thermo Fisher Scientific, Bremen, Germany) was used to identify the lipid profile of the obtained *N. oceanica* and *C. amblystomatis* extracts [32] (Supplementary Table S1).

### 2.3. Cell Culturing and Treatment

Human melanoma cell line SK-MEL-28 (HTB-72) obtained from American Type Culture Collection (ATCC, Manassas, VA, USA) were cultured in a two-dimensional model in a humidified atmosphere of 5% CO_2_ at 37 °C in a medium recommended by the manufacturer Eagle’s Minimum Essential Medium (EMEM) supplemented with 10% foetal bovine serum (FBS). To avoid bacteria contamination, 50 U/mL penicillin and 50 μg/mL streptomycin were added to the media. When the cells reached 90% confluence, they were seeded into AlgiMatrix 24-well plates (Life Technologies, Carlsbad, CA, USA) to create a three-dimensional model. The concentration of seeded cells was 5 × 10^5^ per each well. Following four days of incubation, the medium was changed to cold PBS (phosphate-buffered saline, 4 °C), and cells were exposed to the UVA (365 nm) radiation (Bio-Link Crosslinker BLX 365; Vilber Lourmat, Eberhardzell, Germany) in a total dose of 18 J/cm^2^. The dose was selected to approximately 70% of cell viability measured using MTT (3-(4,5-dimethylthiazol-2-yl)-2,5-diphenyltetrazolium bromide) assay [33]. In parallel, cells not exposed to UVA radiation were maintained under the same conditions. Following irradiation, cells were incubated for 24 h in an FBS-free medium supplemented with lipid extract from *Nannochloropsis oceanica* or *Chlorococcum amblystomatis* at a concentration of 3 µg/mL, primarily dissolved in DMSO (dimethyl sulfoxide), which in the final solution was 0.1%. The concentration of extracts was selected based on the previous studies on healthy skin cell lines, in which their biological activity without cytotoxic effect was demonstrated [20,34]. Control cells (non-irradiated or UVA-irradiated) were cultured in parallel in a medium containing 0.1% DMSO.

**Figure 1:**
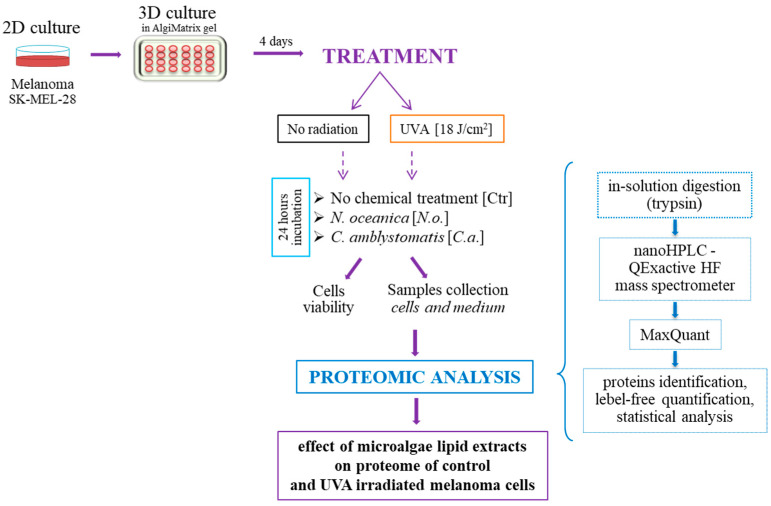
The diagram showing the course of the experiment.

Following incubation, medium was collected and concentrated by centrifugation in Amicon Ultra-0.5 with a cut off at 3 kDa (Merck Milipore; Burlington, MA, USA). The melanoma cells were collected from 3D gel using AlgiMatrix dissolving buffer (Life Technologies, Carlsbad, CA, USA) and lysed by sonification. The diagram showing the whole course of the experiment is shown in Figure 1.

### 2.4. MTT Test

The study of the effects of used microalgae lipid extracts on UVA-exposed melanoma cells proteome was preceded by the verification of the viability of these cells under experimental conditions. For this purpose, MTT assay [33] was performed on cells treated as described above and cultured into AlgiMatrix 96-well plate (5 × 10^3^ cells/well). Following 24 h of experimenting, medium with supplements was removed and MTT solution in PBS (0.25 mg/mL) was added to each well. The plate was incubated for 3 h at 37 °C and 5% CO_2_. Next, the MTT solution was replaced with a DMSO to cells lysis. Absorbance was read at 570 nm on a Multiskan GO microplate spectrophotometer (Thermo Scientific, Waltham, MA, USA). Results were calculated as a percentage of the control sample.

### 2.5. SDS-PAGE-Based Profiling

To analyse the distribution of proteins in cell lysates and medium, their separation was prepared using SDS-PAGE. The total protein concentration in all samples was measured using a Bradford assay [35]. The volume of sample containing 25 μg of protein was denatured using Laemmle buffer with 5% of 2-mercaptoethanol at 100 °C for 7 min. Next, the proteins were separated on 10% Tris-Glycine SDS-PAGE gels. Following fixing (1 h in 5:1:4 of methanol: acetic acid: water), gels were stained overnight with Coomassie Brilliant Blue R-250. Indicated bands were documented using the Versa Doc System and Quantity One 4.6.9 software (Bio-Rad Laboratories Inc., Hercules, CA, USA) and are presented in Supplementary Figure S1.

### 2.6. Protein Digestion and Peptide Analysis

Samples containing 50 µg proteins were denatured by 8 M urea, reduced with 10 mM 1,4-dithiothreitol (DTT), and alkylated with 50 mM iodoacetamide (IAA). Before digestion, samples were fourfold diluted with ammonium bicarbonate buffer (AMBIC, 25 mM). Proteins were digested in solution overnight (37 °C) with trypsin (1:50 of trypsin:protein) (Promega, Madison, WI, USA). To stop the reaction, 10% formic acid (FA) was added (final concentration of FA was 0.1%) [36] and samples were dried under inert gas. Partial data validation the experiment was also repeated with the use of in-gel protein digestion as described before [37]. 

Peptides obtained after digestion were dissolved in 5% acetonitrile (ACN) with 0.1% FA and separated using the high-performance liquid chromatography system Ultimate 3000 (Dionex, Idstein, Germany) with a 50 mm × 75 µm PepMap RSLC capillary analytical C18 column (Dionex LC Packings, Dionex, Idstein, Germany) at a flow rate of 0.300 µL/min. Eluted peptides were analysed using a Q Exactive HF mass spectrometer with an electrospray ionisation source (ESI) (Thermo Fisher Scientific, Bremen, Germany). The mass spectrometer was operated in a positive mode. The resolution of 120,000 was applied for MS scan analysis and 30,000 for MS/MS. The Q Exactive mass spectrometer was operated using Xcalibur 4.1 (Thermo Fisher Scientific, Bremen, Germany). Conditions of the peptide analysis have been described in detail previously [38].

### 2.7. Protein Identification and Label-Free Quantification

MaxQuant v2.4.2 software was used to analyse raw data [39] against the UniProtKB-SwissProt database (taxonomy: Homo sapiens, release September 2023). As a dynamic modification of the chosen amino acids (cysteine, lysine, and histidine), adducts formation with 4-hydroxynonenal (4-HNE) or 15-prostaglandin J2 (15-PGJ2) was set, according to the Unimod Protein Modifications for Mass Spectrometry Database [40,41]. The signal intensities of the precursor ions were used for label-free quantification of proteins. The modified protein quantification was conducted based on the peak area. Only proteins with at least three identified peptides longer than 6 amino acid residues and at least two unique peptides were taken for statistical analysis.

### 2.8. Statistical Analysis

Each cell variant was repeated in five independent biological replicates. The results from individual protein label-free quantification were subjected to data imputation (missing values were replaced by 1/10 of the minimal positive values of their corresponding variables), normalised by the sum of the protein intensities, log-transformed, and normalised with EigenMS [42]. Data were tested for homogeneity of variance and normal distribution using the open-source software RStudio (2024.09.1+394) [43]. MetaboAnalyst 5.0 (http://www.metaboanalyst.ca; accessed on 11 December 2023) [44] was used for biostatistical analysis, including univariate analysis one-way (ANOVA, Fisher’s least significant differences (LSD), the false discovery rate (FDR) < 5%), heatmaps, and dendrogram creation. Results that show low repeatability (RDS (relative standard deviation) more than 20%) were removed from the subsequent analysis. Protein functions were determined using Protein Analysis Through Evolutionary Relationships Classification System (PANTHER 17.0) [45].

## 3. Results

**Figure 2:**
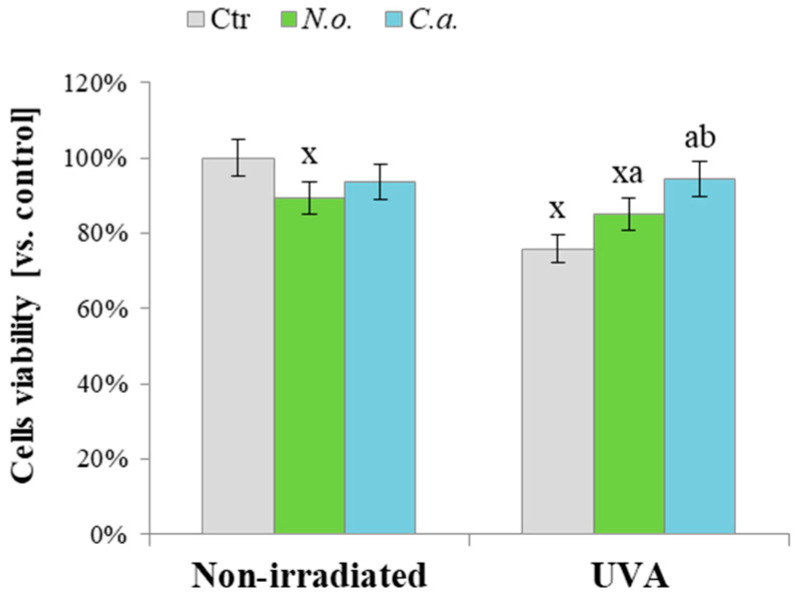
The viability of control (Ctr) and UVA (18 J/cm^2^) irradiated melanoma cells treated with algae lipid extracts (3 ng/mL; *N.o.*, *Nannochloropsis oceanica*; *C.a.*, *Chlorococcum amblystomatis*) cultured in vitro in a three-dimensional (3D) model was measured using the MTT assay. The results are presented as mean values ± standard deviation (SD) with statistically significant differences (*p* < 0.05) indicated as follows: x—vs. Ctr cells; a—vs. UVA irradiated cells; b—vs. *N.o.* non-irradiated/irradiated cells, respectively.

Results from this study indicate that bioactive compounds contained in the lipid extracts of microalgae *Nannochloropsis oceanica* (*N.o.*) and *Chlorococcum amblystomatis* (*C.a.*) can significantly influence the metabolism of 3D-cultured melanoma cells, including those subjected to UVA irradiation, as observed even at the viability level (Figure 2). UVA radiation reduced cell viability to 70% of the control group. However, treatment with microalgae extracts mitigated these effects; in the case of *N.o.*, UVA-induced changes were reversed by 15%, and for *C.a.*, by 25%, restoring cell viability to the level of the control group.

Proteomic analysis identified and quantified 1240 proteins in cell lysates and 128 in the medium of experimental cultures, all meeting the statistical requirements for analysis (Supplementary Table S2). In both cell lysates and medium, the total detected missing values were no more than 0.2% of all quantified proteins.

**Figure 3:**
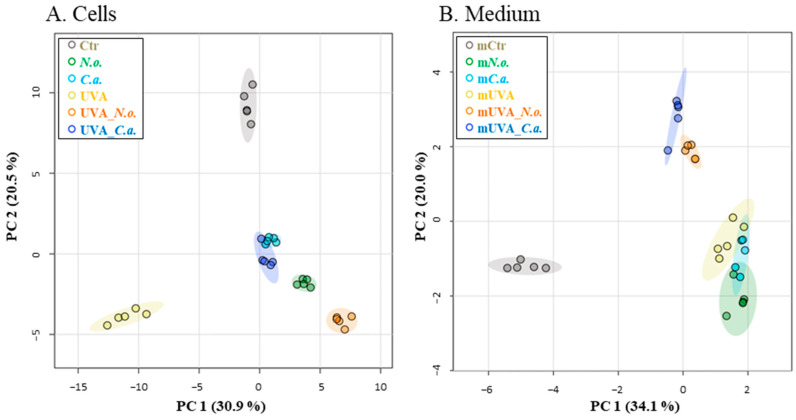
Principal component analysis (PCA) was performed to assess protein expression in control (Ctr) and UVA (18 J/cm^2^) irradiated melanoma cells treated with algae lipid extracts (3 ng/mL; *N.o.*, and *Nannochloropsis oceanica*; and *C.a.*, *Chlorococcum amblystomatis*) cultured in vitro in a three-dimensional (3D) model. The results obtained for cell lysates are shown in (**A**), and for FBS-free medium, labelled with “m”, in (**B**).

Preliminary in-gel protein separation did not reveal significant differences in the band distribution in cells, but notable differences were observed in the medium, particularly in the region of proteins with molecular weight 40–70 KDa (Supplementary Figure S1). The proteins identified in this size band as the most intense included epidermal growth factor receptor (EGFR, P00533), protein disulfide-isomerase A4 (PDIA4, P13667), fibroblast growth factor 2 (FGF 2, P48798), and protransforming growth factor β3 (TGF β3, P10600). Moreover, the proteomic analysis further differentiated between the melanoma cells treated in various ways. Principal component analysis (PCA) separated UVA-irradiated cells along the horizontal plane (PC1-30.9%) and control cells along the vertical plane (PC2-20.5%), while the profiles of cells treated with algae extracts, with or without UVA irradiation, were closer to each other (Figure 3A). These findings are supported by dendrogram analysis, which showed significant differentiation between UVA-irradiated cells and other treated/control cells. Control cells were centrally located on the dendrogram arms, while cells treated with algae extracts, especially *C.a*. (with or without UVA irradiation), formed a closely grouped cluster (Supplementary Figure S2A). The medium presented slightly different results, with control samples horizontally distanced from the rest (PC1-34.1%), and samples treated with *N.o.* or *C.a.* following UVA irradiation separated vertically from the remaining samples (PC2-20.0%) (Figure 3B). The dendrogram also showed that the medium from control cells formed a separate branch distinct from the profiles of other samples (Supplementary Figure S2B).

**Figure 4:**
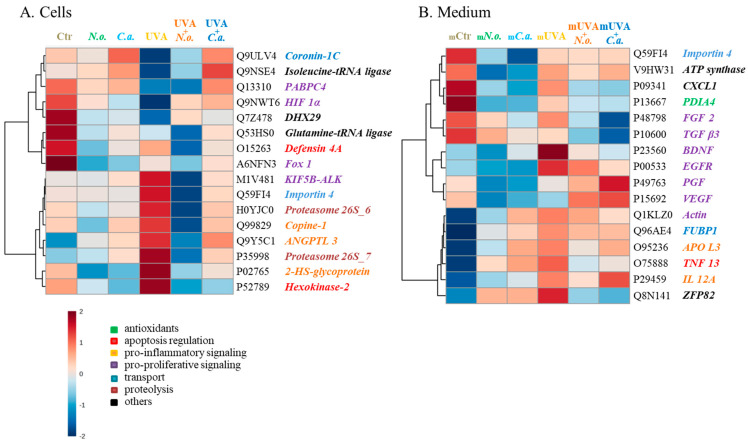
Heatmap and clustering of the top 16 proteins, with the lowest *p*-values, in control (Ctr) and UVA (18 J/cm^2^) irradiated melanoma cells treated with algae lipid extracts (3 ng/mL; *N.o.*, *Nannochloropsis oceanica*; and *C.a.*, *Chlorococcum amblystomatis*) cultured in vitro in a three-dimensional model (3D). Results are shown for cell lysates (**A**) and FBS-free medium (**B**). Protein abbreviations are as follows: ANGPTL, angiopoietin; APO, apolipoprotein; BDNF, brain-derived neurotrophic factor; CXCL1, growth-regulated alpha protein; DHX29, ATP-dependent RNA helicase; EGFR, epidermal growth factor receptor; FGF, fibroblast growth factor; Fox, forkhead box protein; FUBP1, Far upstream element-binding protein 1; HIF, hypoxia-inducible factor; IL, interleukin; KIF5B-ALK, tyrosine-protein kinase receptor; PABPC4, polyadenylate-binding protein 4; PDIA4, protein disulfide-isomerase A4; PGF, placenta growth factor; TGF, protransforming growth factor; TNF, tumour necrosis factor; VEGF, vascular endothelial growth factor; and ZFP, zinc finger protein.

**Figure 5:**
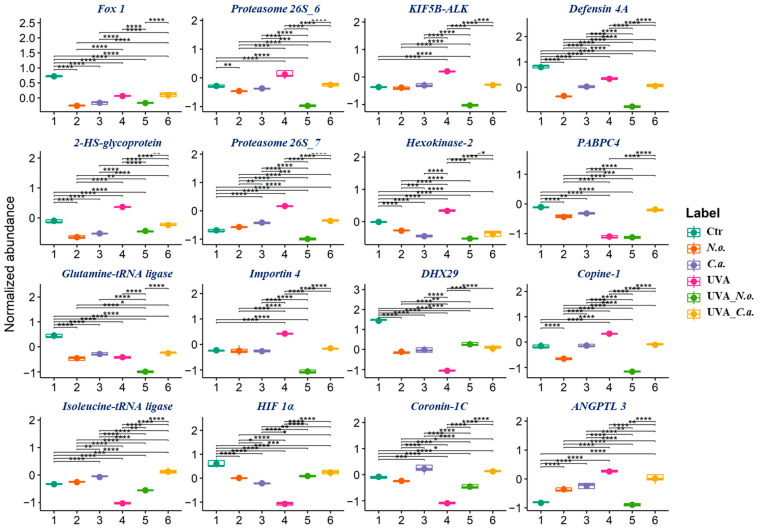
Boxplots of the top 16 proteins, with the lowest *p*-values, in control (Ctr) and UVA (18 J/cm^2^)-irradiated melanoma cells treated with algae lipid extracts (3 ng/mL; *N.o.*, *Nannochloropsis oceanica*; and *C.a.*, *Chlorococcum amblystomatis*) cultured in vitro in a three-dimensional model (3D). Protein abbreviations are as follows: ANGPTL, angiopoietin; DHX29, ATP-dependent RNA helicase; Fox, forkhead box protein; HIF, hypoxia-inducible factor; KIF5B-ALK, tyrosine-protein kinase receptor; and PABPC4, polyadenylate-binding protein 4. Statistically significant differences are marked as follows: *, *p* < 0.05; **, *p* < 0.01; ***, *p* < 0.001; ****, and *p* < 0.0001.

The list of the top 16 modified proteins, with the lowest *p*-values, revealed that the proteins most influential in separating experimental groups in cell samples were involved in pro-inflammatory signalling (copine-1 (Q99829), angiopoietin 4 (ANGPTL-4, Q9Y5C1), and 2-HS-glycoprotein (P02765)), apoptosis regulation (defensin 4A (O15263), hexokinase-2 (P52789)), proteolysis (proteasome 26S subunits 6 and 7 (H0YJC0, P35998)), and gene expression and cell proliferation (polyadenylate-binding protein 4 (PABPC4, Q13310), hypoxia-inducible factor 1-alpha (HIF 1α, Q9NWT6), forkhead box 1 protein (Fox 1, A6NFN3), and tyrosine-protein kinase receptor (KIF5B-ALK, M1V481)) (Figure 4A). Results show that in control melanoma cells, the most abundant proteins were those responsible for apoptosis regulation, gene expression, and cell proliferation, while UVA radiation significantly increased pro-inflammatory proteins and decreased levels of proteins involved in gene expression and cell proliferation. The algae extracts significantly reduced these protein levels in non-irradiated cells and after irradiation, but post-irradiation differences between the two algae-treated cells were more pronounced: in the UVA + *C.a.* group, UV-induced changes were more substantially elevated than in the UVA + *N.o.* group (Figure 5). 

**Figure 6:**
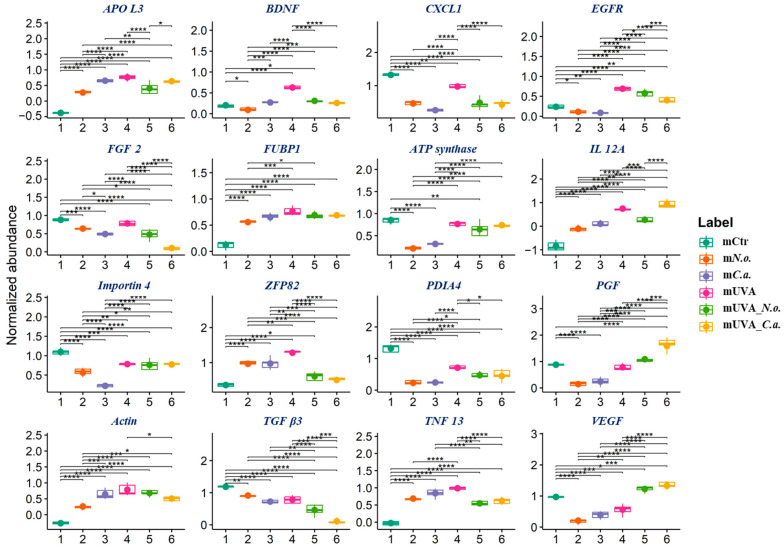
Boxplots of the top 16 proteins, with the lowest *p*-values, in the medium in control (Ctr) and UVA (18 J/cm^2^)-irradiated melanoma cells treated with algae lipid extracts (3 ng/mL; *N.o.*, *Nannochloropsis oceanica*; and *C.a.*, *Chlorococcum amblystomatis*) cultured in vitro in a three-dimensional model (3D). Protein abbreviations are as follows: APO, apolipoprotein; BDNF, brain-derived neurotrophic factor; CXCL1, growth-regulated alpha protein; EGFR, epidermal growth factor receptor; FGF, fibroblast growth factor; FUBP1, Far upstream element-binding protein 1; IL, interleukin; PDIA4, protein disulfide-isomerase A4; PGF, placenta growth factor; TGF, protransforming growth factor; TNF, tumour necrosis factor; VEGF, vascular endothelial growth factor; and ZFP, zinc finger protein. Statistically significant differences are marked as follows: *, *p* < 0.05; **, *p* < 0.01; ***, *p* < 0.001; ****, *p* < 0.0001.

In the medium collected from these cells, the top 16 modified proteins, with the lowest *p*-values, were involved in the induction of cell growth and proliferation (fibroblast growth factor 2 (FGF 2, P48798), protransforming growth factor β3 (TGF β3, P10600), brain-derived neurotrophic factor (BDNF, P23560), epidermal growth factor receptor (EGFR, P00533), placenta growth factor (PGF, P49763), vascular endothelial growth factor (VEGF, P15692), actin (Q1KLZ0), growth-regulated alpha protein (CXCL1, P09341)), pro-inflammatory signalling (interleukin 12A (IL-12A, P29459), apolipoprotein L3 (APO L3, O95236)), transport (importin 4 (Q59FI4), Far upstream element-binding protein 1 (FUBP1, Q96AE4)), apoptosis (TNF 13 (O75888)), and antioxidant response (protein disulfide-isomerase A4 (PDIA4, P13667)) (Figure 4B). Control cells released to the medium a significant amount of proteins responsible for cell growth and proliferation, a process that UVA radiation inhibited by promoting the outflow of pro-inflammatory molecules. The application of algae extracts decreased the release of pro-proliferative molecules and mildly increased pro-inflammatory signalling (Figure 6).

**Figure 7:**
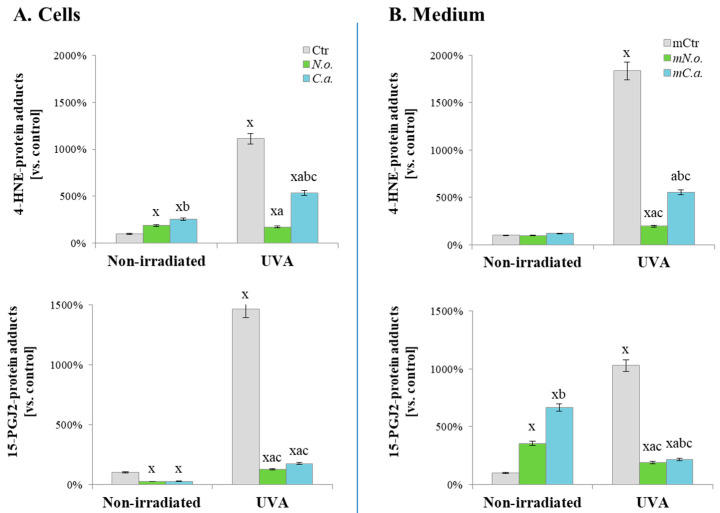
Total levels of protein modifications by lipid peroxidation products (4-hydroxynonenal (4-HNE) and 15-deoxy-12,14-prostaglandin J2 (15d-PGJ2)) in control (Ctr) and UVA (18 J/cm^2^) irradiated melanoma cells treated with algae lipid extracts (3 ng/mL; *N.o.*, *Nannochloropsis oceanica*; *C.a.*, *Chlorococcum amblystomatis*) cultured in vitro in a three-dimensional model (3D). Results were obtained for cell lysates (**A**) and FBS-free medium (**B**). Mean values ± SD are presented with statistically significant differences (*p* < 0.05): x—vs. Ctr cells; a—vs. UVA irradiated cells; b—vs. *N.o.* non-irradiated/irradiated cells, respectively; and c—vs. non-irradiated, *N.o.*/*C.a.*-treated cells, respectively.

Despite these changes in protein expression, UVA radiation and algae extracts also induced structural changes in proteins by modifying them with products of lipid metabolism (Figure 7). UVA was the primary factor that induced these modifications in both cells and medium. Treatment with algae extracts led to the generation of 4-HNE-protein adducts but decreased the level of 15-PGJ2-protein adducts. After exposure to UVA, both extracts effectively prevented the high level of these modifications (Figure 7A). In the medium of the experimental cells, algal extracts did not affect the formation of 4-HNE-protein adducts, but significantly induced the formation of protein adducts with 15-PGJ2. Following exposure to radiation, the level of both types of protein modifications was reduced by up to tenfold by the algal extracts (Figure 7B). Furthermore, comparing the effects of the extracts, the extract from *Nannochloropsis oceanica* was more than three times as effective as the extract from *Chlorococcum amblystomatis* at reducing the UVA-induced increase in 4-HNE-protein adducts in both cells and medium. This effect was also evident in the case of 15-PGJ2-protein adducts.

## 4. Discussion

Given the multidirectional metabolic changes occurring during cancer development, comprehensive studies using omics techniques, which capture a broader range of changes than traditional quantitative methods, are increasingly warranted [46]. Furthermore, in experimental research that relies solely on cell cultures, the use of an appropriate model that closely reflects potential cell interactions, as well as the analysis of factors that not only assess the functioning of cancer cells but also their impact on neighbouring cells and tissues, is crucial [47]. In this study, we assessed the proteomic changes in 3D-cultured melanoma cells subjected to UVA radiation stress and evaluated the changes in the protein profiles released by these cells into the medium. Additionally, this experiment explored the impact of lipid extracts from the microalgae *Nannochloropsis oceanica* and *Chlorococcum amblystomatis* on these changes.

### 4.1. The Effect of UVA on Melanoma Cell Proteome and Released Factors

UVA radiation represents one of the primary harmful physical factors to which human skin cells are exposed daily. In the context of melanoma cells, this radiation is doubly significant because it induces oxidative stress and damage to intracellular structures, and it promotes the formation of cancerous lesions [48,49]. Given that UVA is considered a primary cause of melanoma, much research focused on the role of UVA in cancer transformation [50], although its effects on melanoma cells are often overlooked. The results presented in this study clearly demonstrate that UVA radiation induces significant changes in the proteome of melanoma cells, notably the increased expression of pro-inflammatory and pro-proteolytic proteins, which increased dramatically following UVA exposure compared to non-irradiated cells. This response was likely due to the damaging effects of UVA radiation, which naturally triggers inflammatory processes in skin cells [51,52]. Additionally, UVA radiation leads to oxidative metabolism of cellular membrane lipids, resulting in the production of lipid peroxidation products such as reactive aldehydes, and through enhanced enzymatic activity, the generation of lipid mediators, including eicosanoids and prostaglandins, which are involved in pro-inflammatory signalling [53]. Consequently, this treatment induced the biosynthesis of pro-inflammatory cytokines, including IFN-γ, interleukins, and TNF-α, which are essential for proper acute inflammation in the skin, facilitating the infiltration of immune cells to eliminate cells damaged by UV exposure [54].

Simultaneously, the proteomic analysis of the medium showed that UVA radiation also altered the profile of proteins released by melanoma cells. The observed increase in pro-inflammatory factors in the medium aligns with the discussed pro-inflammatory effects of UVA radiation. Additionally, UVA radiation decreased the level of excreted growth factors, such as TGFs, FGFs, and PGF, which are biosynthesised and released by melanoma cells, thus slowing their proliferation [55,56], likely in response to the harmful pro-oxidant conditions induced by UVA radiation. While most of these factors are known to be upregulated by UV radiation in many cell lines, including skin cells [57,58,59,60], there is limited data on their release outside the cell, which requires appropriate mechanisms for crossing the UV-modified membrane [61].

Beyond direct changes in protein expression, UVA radiation also altered protein structure through modification with products of lipid metabolism: 4-HNE (a product of lipid peroxidation) and the eicosanoid 15-PGJ2, observed in both cells and the medium. Results indicate that in the medium, the UVA-induced increase in 4-HNE-protein adducts was more pronounced than in cells, whereas the opposite is observed for 15-PGJ2-protein adducts. Both 4-HNE and 15-PGJ2 play roles in intracellular and extracellular stress-response signalling, although their ranges of action differ; 4-HNE, in both its free and protein-bound forms, primarily activates the antioxidant system, while 15-PGJ2 regulates the cell cycle, growth, proliferation, and apoptosis [62,63]. A common feature in their actions is their effect on the inflammatory response, particularly through their interactions with proteins. 4-HNE can inhibit pro-inflammatory signalling both by inhibiting NFκB and activating the antioxidant system, while 15-PGJ2 has a similar effect through the formation of adducts with IκB and STAT3 and interactions with specific receptors, including PPARs [64,65]. Thus, the robust formation of adducts in UVA-irradiated melanoma cells is seen as a defence against the induction of pro-inflammatory signalling, a mechanism that has been widely described but previously did not include signalling based on lipid peroxidation products [66], a novel finding of this study.

### 4.2. The Effect of Microalgae Extracts on Melanoma Cells

*Nannochloropsis oceanica* and *Chlorococcum amblystomatis* are garnering increasing attention for potential uses in the food industry, energy, cosmetology, and medicine due to their therapeutic effects. They are rich in essential amino acids (13–40% of dry weight biomass), vitamins, minerals, pigments (1–15%), and bioactive lipids (9–32%), particularly PUFAs. Among these, phospholipids, the primary carriers of omega-3 fatty acids, and glycolipids are noted for their significant bioactive properties [32,67]. Their metabolic products are known for their antioxidant, anti-inflammatory, anti-obesity, anti-tumour, and anti-microbial/viral properties [68]. Previous studies have shown that extracts from *Nannochloropsis oceanica* are non-toxic to skin cells such as keratinocytes and fibroblasts [20,69], a finding similarly suggested for *Chlorococcum amblystomatis* [unpublished data]. However, the results presented here indicate that these extracts slightly reduce the viability of non-irradiated melanoma cells, particularly in the case of *Nannochloropsis oceanica*, confirming the anti-cancer properties of these extracts [21,22,23,24]. This effect is not readily apparent in proteomic changes within the cells, likely due to the strong antioxidant and anti-inflammatory properties of these algae extracts. The list of the top 16 modified proteins (with the lowest *p*-values) indicates that both extracts reduce levels of pro-proliferative proteins as well as pro-inflammatory molecules (Figure 4 and Figure 5). Moreover, the results reveal differences in the action of the two algae extracts; the extract from *Nannochloropsis oceanica* more significantly alters the profile of melanoma cells than the extract from *Chlorococcum amblystomatis*. This may be attributed to the stronger DPPH• radical scavenging properties of *Nannochloropsis oceanica* compared to *Chlorococcum amblystomatis* (inhibitory concentration ratio IC15:IC40, respectively) [32,67]. Additionally, observed differences may relate to significant variations in extract composition, particularly in the total level of saturated fatty acids (SFA). The SFA content in the *Nannochloropsis oceanica* extract is about 80% lower than that in *Chlorococcum amblystomatis* [32,67]. Importantly, the higher SFA intake has been associated with enhanced inflammation and increased generation of pro-inflammatory cytokines [70].

On the other hand, special attention should be given to the changes induced by the extracts in the profile of released proteins. Both extracts reduce the levels of released growth factors, a change that parallels those induced by UVA radiation, which also produces an anti-cancer effect. Additionally, the pro-inflammatory action of algae extracts in the context of melanoma cells may be related to the specifically intensified oxidative metabolism of fatty acids in these cells [71,72]. Moreover, the extract of *Nannochloropsis oceanica* induces more pronounced changes than the extract of *Chlorococcum amblystomatis* in the expression of pro-inflammatory proteins. This observation might be related to the higher concentration of main fatty acids in the *Nannochloropsis oceanica* extract, such as C16:1, C18:1, C20:4, and C20:5 compared to *Chlorococcum amblystomatis* [32,67]. Consequently, supplementation of melanoma cells with these compounds may promote the generation of metabolism products characterised by the ability to induce pro-inflammatory signalling [73,74] and increase the release of pro-inflammatory proteins outside the cell. In vivo, this may result in two different effects: firstly, the emerging inflammatory microenvironment is associated with the influx of cytotoxic T cells and T helper cells, which confer antitumor immunity and a good prognosis for patients with cancer; secondly, pro-inflammatory mediators released by cancer cells induce several molecular signalling cascades in adjacent healthy cells, including MAPK, PI3K, Nrf2 (nuclear factor erythroid 2-related factor 2), or Janus kinases/STAT, which generally promote their antioxidant activity and increase proliferation [75].

Algae extracts also modify the level of 4-HNE and 15-PGJ2-protein adducts in cells and medium. As previously mentioned, 4-HNE protein adducts are crucial in inducing the antioxidant response [76]; therefore, their increased formation in melanoma cells corresponds with the increased level of antioxidant enzymes, also induced by these extracts. On the other hand, algae extracts induce varied changes in the case of 15-PGJ2-protein adducts. The decreased level of these modifications in melanoma cells may be the reason for the lack of silencing of pro-inflammatory pathways [77], which is also evidenced by the increased level of pro-inflammatory cytokines (IL-6/12 and ANGPTL 3) compared to untreated cells. Simultaneously, the strong increase in the level of 15-PGJ2-protein adducts in the medium, given their inhibitory role in protein de novo biosynthesis, cell growth, and proliferation [78], along with the decreased level of previously mentioned growth factors, may be the primary reason for the observed decrease in melanoma cell viability. Moreover, the release of 15-PGJ2-protein adducts by treated cancer cells as signalling molecules in vivo may play a promising role in anticancer therapy because these factors activate the Keap1/Nrf2 pathway, reduce oxidative stress, and by stabilizing oxygen metabolism and limiting the proliferation of endothelial cells, they prevent tumour-induced angiogenesis [79,80].

### 4.3. The Effect of Microalgae Extracts on UVA-Irradiated Melanoma Cells

The effects of lipid extracts from microalgae on melanoma cells may have a positive impact during anticancer therapy. This experiment revealed the effects of these extracts on cells after exposure to UVA radiation. Their strong antioxidant properties, by reducing stress caused by UVA radiation, partially prevented cell mortality, slowing their death and the uncontrolled release of toxic products from undigested cells into the surroundings. This action may also be based on the reduction in inflammatory processes observed here as a decreased level of pro-inflammatory proteins. This is significant because by reducing inflammatory processes, the extracts enable cancer cells to enter the path of apoptosis without the risk of necrosis and additional inflammatory reactions [81]. Moreover, the results show significant differences in the effects of the two algae extracts following cell irradiation; the extract of *Nannochloropsis oceanica* reduced UV-induced changes more strongly than the extract of *Chlorococcum amblystomatis*. These observations focus mainly on the regulation proteins of inflammation and apoptosis. One of the proteins that combine both functions is defensin 4A, found in most cancer cells and described as activating the adaptive immune system with the generation of anti-tumour immunity [82]. Additionally, defensin can inflict DNA damage and induce apoptosis of tumour cells, thus also revealing pro-apoptotic effects [82]. Furthermore, the extract of *Nannochloropsis oceanica* induces a stronger protective effect than the extract of *Chlorococcum amblystomatis*, which greatly differentiates the profile of treated melanoma cells from untreated cells, suggesting an unknown mechanism of action of these extracts or their potential undesirable effects, especially in cells following exposure to UVA radiation.

The extracts of *Nannochloropsis oceanica* and *Chlorococcum amblystomatis* modified the profile of proteins released from UVA-irradiated melanoma cells in varying degrees but in a similar direction. The significant decrease in 5 of 7 detected growth factors indicates their role in silencing the growth and proliferation of melanoma post-UVA irradiation observed in this study. However, the influence of the extracts on the remaining two factors (PGF, VEGF) is unknown. Moreover, the extracts strongly induced the level of released interleukin IL-12A in UVA-irradiated melanoma cells, potentially leading in vivo to the influx of phagocytic cells [83,84]. Additionally, lipid extracts reduced the level of 4-HNE and 15-PGJ2-protein adducts similarly in both cells and the medium. This might be related to their antioxidant properties [32,67], which, as with other antioxidants, reduce the pro-oxidative effect of UVA radiation and UVA-induced lipid metabolism [85].

## 5. Conclusions

The high mortality of melanoma patients, along with the rapid growth rate of melanoma cells, requires the continual search for effective and safe methods to enhance anti-tumour therapy. The results obtained in this study suggest that lipid extracts of *Nannochloropsis oceanica* and *Chlorococcum amblystomatis*, given their anti-proliferative properties, should be considered in this context. However, their strong protective capacity may limit the scope of their use, especially in the case of UVA-irradiated cells. UVA is a factor that affects all skin cells in vivo, including melanoma cells, while supplementing them with microalgae extracts, particularly the extract of *Nannochloropsis oceanica*, post-irradiation supports melanoma cell defence against apoptosis, thus promoting survival, drug resistance, and metastasis of the cancer.

## Data Availability

The authors confirm that the data supporting the findings of this study are available within the article and its supplementary materials.

## Supplementary Materials

The following supporting information can be downloaded at: https://www.mdpi.com/article/10.3390/cells13231934/s1, Supplementary Table S1. The lipid profile of the obtained *Nannochloropsis oceanica* and *Chlorococcum amblystomatis* extracts characteried by hydrophilic interaction liquid chromatography coupled with high-resolution mass spectrometry (HILIC-MS) and tandem MS (MS/MS) using a Q-Exactive hybrid quadrupole Orbitrap mass spectrometer (Thermo Fisher Scientific, Bremen, Germany). Supplementary Table S2. The IDs of the proteins that were identified and semi-quantified in melanoma cells cultured in vitro in a three-dimensional (3D) and medium of experimental cultures after irradiation with UVA (18 mJ/cm^2^) and 24 h incubation with a lipid extracts (3 ng/mL) of microalgae *Nannochloropsis oceanica* and *Chlorococcum amblystomatis*. Data obtained using high-performance liquid chromatography system Ultimate 3000 (Dionex, Idstein, Germany) coupled Q Exactive HF mass spectrometer (Thermo Fisher Scientific, Bremen, Germany). Raw data were analysed by the MaxQuant v2.4.2 using the UniProtKB-SwissProt database (taxonomy: Homo sapiens, release September 2023). Supplementary Figure S1. Preliminary protein separation of control (Ctr) and UVA (18 J/cm^2^) irradiated melanoma cells treated with algae lipid extracts (3 ng/mL; *N.o.*, *Nannochloropsis oceanica*; *C.a.*, *Chlorococcum amblystomatis*) cultured in vitro in a three-dimensional (3D) model. Results obtained from cell lysates (A) and FBS-free medium (B, samples labelled with “m”) following SDS-PAGE and Coomassie staining are shown. The arrows on the right highlight the areas with the most significant differences between samples. Supplementary Figure S2. The dendrogram and clustering analysis of control (Ctr) and UVA (18 J/cm^2^) irradiated melanoma cells treated with algae lipid extracts (3 ng/mL; *N.o.*, *Nannochloropsis oceanica; C.a.*, *Chlorococcum amblystomatis*) cultured in vitro in a three-dimensional model (3D). The results obtained for cell lysates are shown in panel A and FBS-free medium in panel B.
