# Supplementary material for: Impact of Nannochloropsis oceanica and Chlorococcum amblystomatis Extracts on UVA-Irradiated on 3D Cultured Melanoma Cells: A Proteomic Insight
Source: Cells. 2024 Nov 21;13(23):1934. doi: 10.3390/cells13231934 (PMC11640244; doi:10.3390/cells13231934)

**Supplementary Figure S1**

Preliminary protein separation of control (Ctr) and UVA (18 J/cm$^2$) irradiated melanoma cells treated with algae lipid extracts (3 ng/mL; N.o., *Nannochloropsis oceanica*; C.a., *Chlorococcum amblystomatis*) cultured in vitro in a three-dimensional (3D) model. Results obtained from cell lysates (A) and FBS-free medium (B, samples labelled with "m") following SDS-PAGE and Coomassie staining are shown. The arrows on the right highlight the areas with the most significant differences between samples.

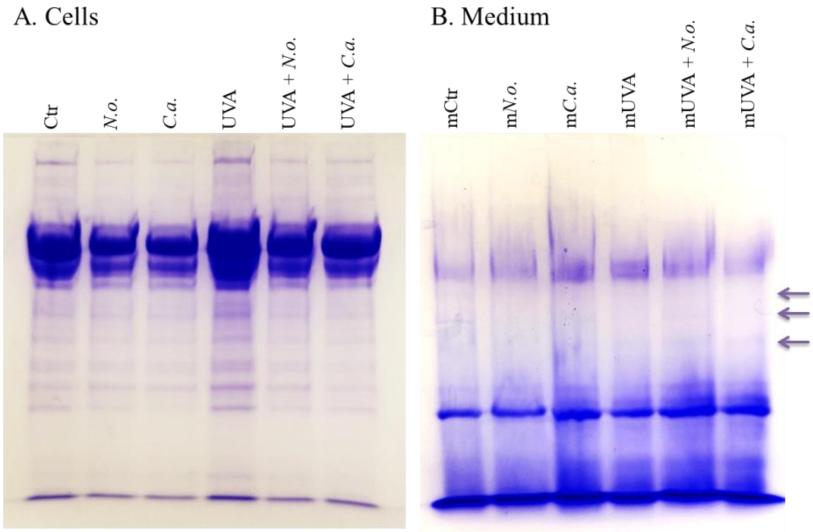

Supplement: Supplementary file 1 [file cells-13-01934-s001.zip › Supplementary Figure S1.pdf]
