# Supplementary material for: Impact of Nannochloropsis oceanica and Chlorococcum amblystomatis Extracts on UVA-Irradiated on 3D Cultured Melanoma Cells: A Proteomic Insight
Source: Cells. 2024 Nov 21;13(23):1934. doi: 10.3390/cells13231934 (PMC11640244; doi:10.3390/cells13231934)

Supplementary Figure S2

The dendrogram and clustering analysis of control (Ctr) and UVA (18 J/cm^2) irradiated melanoma cells treated with algae lipid extracts (3 ng/mL; *N.o., Nannochloropsis oceanica*; *C.a., Chlorococcum amblystomatis*) cultured *in vitro* in a three-dimensional model (3D). The results obtained for cell lysates are shown in panel A and FBS-free medium in panel B.

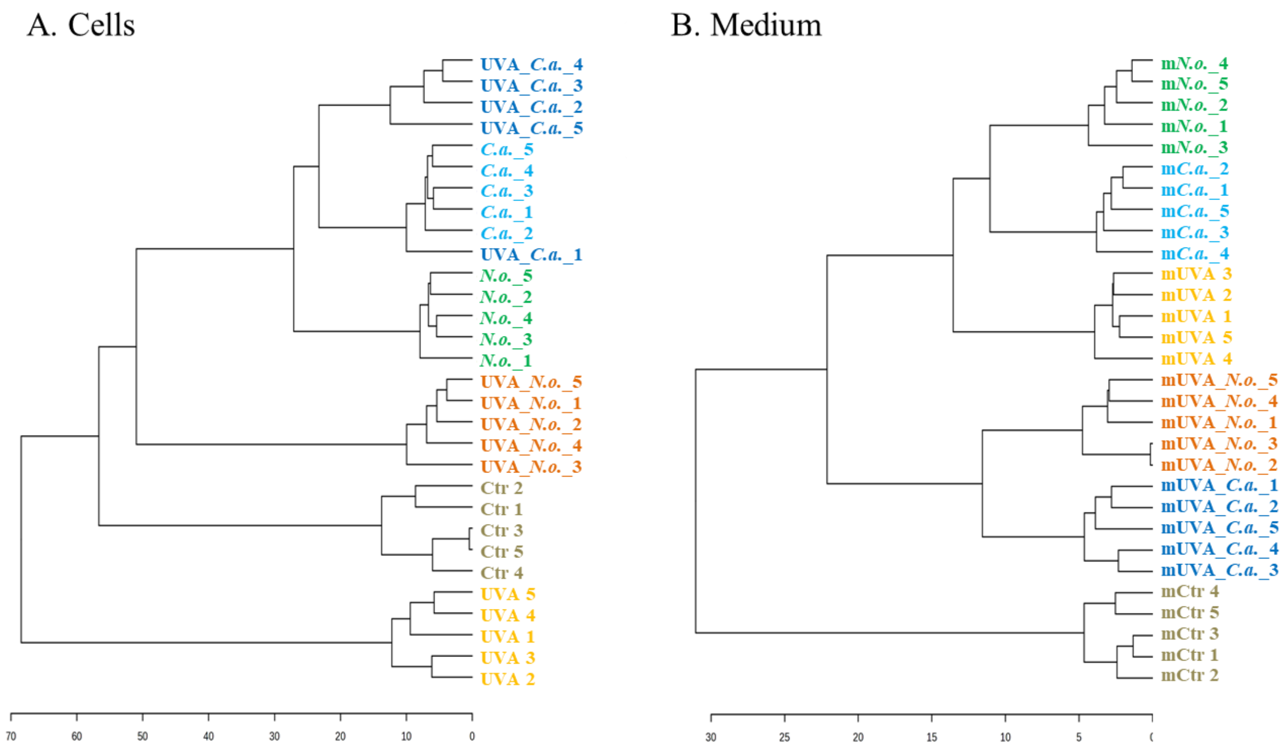

Supplement: Supplementary file 1 [file cells-13-01934-s001.zip › Supplementary Figure S2.pdf]
